# Iron EDTA ingestion and toxicosis in 61 dogs: a multicenter retrospective study of Australian hospital records

**DOI:** 10.3389/fvets.2025.1622800

**Published:** 2025-10-01

**Authors:** Elvany Luswanto, Claire R. Sharp, Erin Mooney, Sarah L. Purcell

**Affiliations:** ^1^School of Veterinary Medicine, Murdoch University, Murdoch, WA, Australia; ^2^Harry Butler Institute, Centre for Terrestrial Ecosystem Science and Sustainability, Murdoch University, Murdoch, WA, Australia; ^3^Faculty of Science, Sydney School of Veterinary Science, The University of Sydney, Camperdown, NSW, Australia; ^4^Small Animal Specialist Hospital, North Ryde, NSW, Australia; ^5^Faculty of Science, School of Veterinary Science, The University of Queensland, Gatton, QLD, Australia

**Keywords:** desferoxamine, vomiting, diarrhea, anaphylaxis, hepatotoxicity, acute respiratory distress syndrome (ARDS), neutropenia

## Abstract

**Introduction:**

There is limited published information on iron toxicosis in dogs, and it is unclear if dogs follow the four stages of progression as in humans. The objective of this retrospective case series was to describe the clinical course and treatment of dogs presenting after iron EDTA ingestion.

**Methods:**

Cases were retrieved through an electronic medical records search at three veterinary teaching hospitals and three private referral hospitals in Australia. Non-parametric descriptive statistics are reported.

**Results:**

61 dogs met the inclusion criteria. The most common iron-containing product ingested was iron EDTA molluscicide (60/61), with only one dog ingesting iron EDTA plant fertilizer. Notably, 20 out of 61 dogs (32.8%) had no clinical signs before presentation. Vomiting without blood was the most common clinical sign (21), followed by lethargy (16), diarrhea without blood (15), ataxia/weakness (12), and signs consistent with abdominal pain (10). Abdominal pain was the most common physical examination finding (21), followed by dehydration (10). Forty dogs underwent some form of gastrointestinal decontamination. The median pre-chelation serum iron concentration was 61.3 μmol/L (Min–Max 9–356, *n* = 40), while the median post-chelation concentration was 14.7 μmol/L (5.72–44, *n* = 22). Overall, 10 dogs were managed as outpatients, while 51 dogs were treated as inpatients. Inpatient treatments included desferoxamine (43), gastroprotectants (41), antiemetics and/or prokinetics (36), analgesia (24), and hepatoprotectants (4). The most common protocol for desferoxamine administration was a continuous intravenous infusion at 15 mg/kg/h (*n* = 27); other dogs received 40 mg/kg intramuscularly (*n* = 13). Two dogs had an anaphylactic reaction to an inadvertent bolus of desferoxamine, while two had allergic reactions possibly related to desferoxamine. Two dogs developed neutropenia, and one developed acute respiratory distress syndrome, possibly as a result of desferoxamine treatment. Overall, 91.8% of dogs survived to discharge. One dog died during hospitalization, experiencing cardiac arrhythmias, shock, and cardiac arrest despite treatment. When considering the stage of iron toxicoses, 16/61 (26.2%) dogs never developed clinical toxicosis, 44 dogs (72.1%) had evidence of Stage 1 clinical signs, 4 dogs (6.6%) also had evidence of Stage 2 clinical signs, and 19 dogs (31.1%) progressed to Stage 3 clinical signs. No dogs had Stage 4 clinical signs (delayed gastrointestinal stricture). A single case was euthanized for acute hepatic failure upon re-presentation after initial survival to discharge.

**Conclusion:**

Iron EDTA toxicosis has a good prognosis in dogs following prompt treatment, but severe organ damage, including hepatic and cardiovascular dysfunction, can occur. Additionally, desferoxamine was well tolerated when administered according to published protocols, but potentially fatal adverse effects can occur.

## 1 Introduction

Iron is an essential element in animal physiology and serves several critical roles. It is vital for oxygen transport, required for many oxidation–reduction reactions, and for the metabolism of many chemicals in the liver, kidneys, and other organs (1, 2). Excess iron in the body, however, can be toxic. Free iron is highly reactive; it can act as and produce free radicals, initiating intracellular oxidative injury that results in severe tissue damage (1, 3).

Iron toxicosis has been reported in domestic animals, including dogs, horses, pigs, and cattle (1). In general, iron damages the gastrointestinal, vascular, hepatic, cardiac, and brain tissues; however, all tissues are susceptible to the toxic effects of free iron (1, 3). Historical reports in humans suggest four stages of toxicity following excessive iron ingestion (4), although the descriptions vary somewhat among sources, and some authors include five stages (5, 6). In the classic four-stage description, the first stage, occurring 0–6 h post-ingestion, is characterized by depression and gastrointestinal effects (nausea, vomiting, diarrhea, and gastrointestinal hemorrhage) due to the direct caustic effects of iron on the gastrointestinal mucosa. Stage 2, 6–24 h post-ingestion, is devoid of clinical signs, and it appears that the patient has recovered. By Stage 3, however, 12–96 h post-ingestion, the most serious clinical signs of systemic toxicosis are evident: lethargy, a recurrence of gastrointestinal signs, metabolic acidosis, hepatic necrosis, cardiovascular collapse, shock, coagulation deficits, and occasionally death. If the patient survives Stage 3, then Stage 4 signs can occur 2–6 weeks post-ingestion, where healing of gastrointestinal ulcerations results in scarring, stricture formation, and gastrointestinal obstruction. Despite these classical descriptions of the toxidrome, a recent report of dogs with iron EDTA molluscicide (“snail pellets”) ingestion described only gastrointestinal toxicity (7).

Dogs are at risk of iron toxicosis due to their indiscriminate eating habits. Sources of iron toxicosis in dogs include iron EDTA or iron phosphate containing molluscicides, iron tablets, iron phosphate fertilizers, iron deoxidizers, and iron-containing hand warmers (1, 3, 8). The frequency of iron ingestion in dogs is not known, but information from animal poison control services suggests that accidental ingestion of iron-containing products is not uncommon (9–12). Despite the reported frequency of exposure, there is very little literature describing iron toxicosis in dogs. To the knowledge of the authors, there are only four peer-reviewed publications describing 140 total cases of accidental iron ingestion and toxicosis in dogs (7, 12–14).

The potential for iron toxicosis after ingestion of an iron-containing substance is based on the dose and form of iron ingested, as well as the rate of absorption. The toxic dose is determined by the amount of elemental iron ingested, regardless of the type of iron salt. To calculate the dose ingested, the amount of iron salt is multiplied by the percentage of elemental iron in that iron salt. For example, iron as ferric salt is 100% elemental iron, while ferric EDTA is 13% elemental iron. Ingestion of 1 gram (g) of ferric salt is therefore equivalent to 1 g of elemental iron, while ingestion of 1 g of ferric EDTA is equivalent to 130 mg of elemental iron. The toxic dose in humans and dogs is reported to start from 20 mg/kg body weight of elemental iron. Mild gastrointestinal upset may occur with ingestion of less than 20 mg/kg of elemental iron (15). At 20–60 mg/kg, mild to moderate intoxication can occur, while ingestion of more than 60 mg/kg of elemental iron can result in severe intoxication. Ingestion of greater than 100–200 mg/kg is potentially lethal (3, 4). Treatment for iron toxicosis focuses on gastrointestinal decontamination, correction of fluid, electrolyte, and acid–base imbalance, and chelation therapy.

Desferoxamine is the most commonly used chelating agent to treat iron toxicosis because of its high affinity for iron (3, 4). Parenterally administered desferoxamine binds free iron and removes iron from transferrin and ferritin, thereby allowing these proteins to bind additional free iron (16). In the existing reports of treating iron toxicosis in dogs with desferoxamine, the most commonly reported dosing protocol is as a continuous rate infusion (CRI) at 15 mg/kg/h (7, 13, 14). When administered by rapid intravenous infusion, desferoxamine can cause arrhythmia and hypotension (4), although only one study reports adverse effects in dogs (7).

The objective of this study was to describe the clinical course of dogs following iron EDTA ingestion, including classification relative to the four stages of toxicosis in humans. A secondary objective was to describe the use of desferoxamine as a chelation agent for the treatment of dogs following iron EDTA ingestion, report the most common dosing protocol used, and any adverse effects following administration. We hypothesized that dogs with iron toxicosis would show the four stages of progression as in humans. Relative to our secondary objective, we hypothesized that dogs with a higher estimated dog of iron ingestion and higher serum iron concentrations would be more likely to receive desferoxamine as a chelation agent.

## 2 Materials and methods

An electronic medical records search was performed at three university veterinary teaching hospitals and three private referral hospitals in Australia. Hospitals included The Animal Hospital at Murdoch University (TAHMU), University of Sydney Veterinary Teaching Hospital, University of Queensland Small Animal Hospital, and three hospitals within the Small Animal Specialist Hospital (SASH) network in New South Wales: North Ryde, Central Coast, and Western Sydney. A fee code search identified cases that had been billed for measurement of serum iron concentration or had desferoxamine prescribed. Species other than dogs, cases where serum iron concentration was measured for reasons other than accidental iron ingestion or suspected iron toxicosis, co-ingestions that could confound assessment of clinical signs, and sources of iron ingestion other than iron EDTA were excluded. Additional cases were captured by searching for the keyword “iron” in dogs presenting to TAHMU between 2010 and 2023. Dogs with highly suspected or confirmed iron EDTA ingestion were eligible for study inclusion after medical record review. Highly suspected or confirmed iron EDTA ingestion was defined by any of the following: owner witnessed ingestion, evidence of chewed up/torn packets of iron-EDTA-containing products, consistent appearance of iron in vomitus, feces, gastric lavage or colonic lavage fluid, evidence of radio-opaque material in the gastrointestinal tract on radiographs after suspected iron ingestion, or an increased pre-chelation serum iron concentration. Since the reference intervals (RIs) varied widely among laboratories measuring iron concentrations, increased serum iron concentration was determined relative to the RI of the laboratory used.

Data extracted from medical records included signalment, body weight (kg), date of presentation, source of iron EDTA exposure, time from ingestion to first presentation, primary accession *versus* referral, type of evidence of ingestion, and clinical signs before presentation to the referral hospital. Additionally, data were collected regarding physical examination findings at the referral hospital, serum iron concentration pre-chelation and/or post-chelation, biochemistry data, type of decontamination performed, success of decontamination, treatments before referral, whether the patient was treated as an inpatient or outpatient, treatments in hospital (for inpatients), treatments at home after discharge, length of hospital stay, complications, and survival to discharge. Dog data were extracted from the medical records and recorded in an online data collection form in REDCap (Research Electronic Data Capture) (Supplementary Data Collection Instrument) (17, 18). After all data entry, a single author (CS) reviewed patient data to categorize each case as to the stage of iron toxicosis based on the aforementioned descriptions in the literature and time course. Cases that only had transient gastrointestinal signs were classified as Stage 1. Those that had clinical signs before presentation but were normal on physical examination upon presentation to the study hospital were classified as having Stage 1 and Stage 2 signs. Those that had gastrointestinal clinical signs before presentation, abnormalities on physical examination, and ongoing clinical signs during hospitalization were classified as Stages 1 and 3.

Age at the time of presentation was calculated within REDCap as the difference between the date of birth and date of presentation in years. Breed was recorded as mixed breed for any dogs described as a cross of one or more breeds. For those dogs that ingested iron EDTA molluscicide, the volume ingested in grams was recorded, if known, allowing calculation of the ingested dose of elemental iron in mg/kg body weight. Iron EDTA is the only active ingredient in iron-containing molluscicides on the market in Australia. The calculation was based on the assumption that there is 7.8 g of elemental iron per kg of molluscicide, as these products contain 60 g/kg of iron EDTA, which is 13% elemental iron (19, 20). When medical records included a range of possible volumes of snail pellets ingested, the larger value was used to represent the maximum possible exposure.

Cases were defined as primary, secondary, or tertiary accession cases depending on whether they presented to other hospital(s) before one of the study hospitals. Information from the medical records was used to estimate the time from ingestion of an iron-containing product to presentation to the first veterinary visit. When a range was noted in the medical record for time from ingestion to presentation, the longest time interval was used for the study. Time from ingestion to presentation was categorized as <1 h, 1 to 2 h, >2 to 6 h, >6 to 12 h, >12 to 24 h, or >24 h.

Physical examination findings considered relevant to iron toxicosis were recorded, including the presence or absence of vasoconstrictive shock, dehydration, pyrexia, abdominal pain, and/or abdominal distention. Abnormal perfusion parameters, including tachycardia, pale mucous membranes, delayed capillary refill time, weak pulses, cool extremities, and decreased mentation, were considered evidence of vasoconstrictive shock (21). Tacky or dry mucous membranes, decreased tear film, and delayed skin tent were considered evidence of dehydration (22). Pyrexia was defined as rectal temperature (T) above 39.1°C (23). Additionally, the administration of an IV fluid bolus or documentation of a rehydration plan for IV fluid therapy was used as evidence of the presence of hypovolemic shock and dehydration, respectively.

Treatment before referral and in-hospital treatment at the study hospital was summarized as to whether the pet underwent decontamination, was treated with IV fluids, or received other medications. Decontamination was defined as successful if the iron-containing product was visually identified in vomitus (following induction of emesis), fluid from gastric or colonic lavage, or enema administration. Colonic lavage was differentiated from simple enema administration if it was performed under general anesthesia. For dogs that were treated with desferoxamine, the dosing regimen and any adverse reactions were also recorded. Medical records were evaluated for information regarding the rationale for stopping desferoxamine therapy (based on urine color, serum iron concentration, an adverse reaction to desferoxamine, or a reason unknown).

For inpatients, length of hospital stay was calculated within REDCap as the difference between the date of admission and date of discharge or non-survival, to the nearest day. As such, an inpatient treated and discharged later on the same calendar day would have had a length of hospital stay of 0 days, even though the patient was admitted and received inpatient care. Outcome was recorded as survived to discharge, died in hospital, euthanized in hospital, or transferred to another veterinary hospital for ongoing inpatient treatment. Dogs that were discharged but later represented to one of the study hospitals were also noted.

### 2.1 Statistical analysis

Descriptive statistical analyses were performed within REDCap (17, 18). Continuous data were assessed for normality by visual inspection of histograms. Given predominantly nonparametric data, continuous variables are described as median (Q1–Q3, Min–Max). Categorical data are described as proportions. Mann–Whitney U tests were used to compare the estimated ingested elemental iron dose and the pre-chelation serum iron concentrations between dogs that received desferoxamine chelation therapy and those that did not. A *p*-value of <0.05 was considered significant.

## 3 Results

Sixty-one dogs with highly suspected or confirmed iron EDTA ingestion, between 2007 and 2023, were included. Of these 61 dogs, 45 were from TAHMU, 10 from one of the SASH hospitals, and 6 from the University of Sydney. Table 1 displays summary statistics for all dogs, as well as dogs categorized by clinical signs. Breeds included mixed breed (16/61, 26.2%) and a variety of pure breeds: notably, Labrador retriever (8, 13.1 %), beagle (4), Border collie (4), two each of American Staffordshire bull terrier, Australian cattle dog, cocker spaniel, Finnish lapphund, and golden retriever. Additionally, 19 additional breeds were represented once.

**Table 1:** Summary statistics for all dogs evaluated for iron EDTA ingestion, as well as dogs categorized by the presence or absence of clinical signs.

Parameter	Cases with no clinical signs at any time (*n* = 16)	Cases with stages 1, 2, and/or 3 toxicosis (*n* = 45)	Summary statistics for all cases (*n* = 61)
Age (years)			
Median	3.21	5.25	5.01
Q1–Q3	2.0–5.18	2.99–8.0	2.56–7.82
Min–Max	0.67–8.58	0.33–16	0.33–16
Sex			
Female spayed	8, 50%	21, 46.7%	29, 47.5%
Male neutered	4, 25%	17, 37.8%	21, 34.4%
Female intact	3, 18.8%	5, 11.1%	8, 13.1%
Male intact	1, 6.3%	2, 4.4%	3, 4.9%
Breed			
Mixed breed	7, 43.8%	9, 20%	16, 26.2%
Pure breeds	9, 56.2%	36, 80%	45, 73.8%
Body weight (kg)			
Median	22.65	18.0	19.1
Q1–Q3	18.28–27.18	13.15–25.8	13.8–26.85
Min–Max	5.7–32.4	3.3–40.1	3.3–40.1
Type of iron-containing product			
Iron EDTA snail pellets	16, 100%	44, 97.8%	60, 98.4%
Iron EDTA fertilizer	0	1, 2.2%	1, 1.6%
Iron dose—iron EDTA snail pellets (mg/kg)			
# with known dose	8	28	36
Median	93.03	155.48	142.9
Q1–Q3	57.02–148.43	109.75–221.19	99.38–216.27
Min–Max	0.37–240.74	31.2–821.05	0.37–821.05
Primary access *vs*. referral			
Primary accession	11, 68.8%	27, 60%	38, 62.3%
Secondary accession	5, 31.2%	16, 35.6%	21, 34.4%
Tertiary accession	0	2, 4.4%	2, 3.3%
Time from ingestion to presentation (hours)			
< 1	3, 18.8%	1, 2.3%	4, 65.5%
1–2	5, 31.3%	3, 6.8%	8, 13.3%
2–6	3, 18.8%	12, 27.3%	15, 25.0%
6–12	2, 12.5%	15, 34.1%	17, 28.3%
12–24	1, 6.3%	11, 25%	12, 20.0%
> 24	2, 12.5%	2, 4.5%	4, 6.7%
Evidence of iron ingestion			
Witnessed ingestion	10, 62.5%	5, 11.1%	15, 24.6%
Empty packet/packet torn/chewed up	5, 31.3%	24, 53.3%	29, 47.5%
Consistent appearance of vomitus or gastric lavage fluid	3, 18.8%	22, 48.9%	28, 45.9%
Iron levels (pre-chelation)	4, 25%	23, 51.1%	27, 44.3%
Consistent appearance of diarrhea, feces on rectal exam, or colonic lavage fluid	3, 18.8%	33, 73.3%	36, 59.0%
Serum iron concentrations (baseline or pre-chelation) μmol/L			
Number, proportion	9	31	40, 65.6%
Median	43.6	65.0	61.3
Q1–Q3	23–71	42.9–72.65	36.73–72.48
Min–Max	9–80	11–356	9–356
Serum iron concentrations (post-chelation) μmol/L			
Number	1	21	22, 36.1%
Median	33	14.0	14.7
Q1–Q3		11.5–22.6	11.63–24.4
Min–Max		5.72–44	5.72–44
Managed as outpatients	7, 43.8%	3, 6.7%	10, 16.4%
Length of hospital stay (days)			
Median	0	1	1
Q1–Q3	0–1	1–2	0–2
Min–Max	0–2	0–4	0–4
Outcome			
Survived to be discharged home	16, 100%	40, 88.9%	56, 91.8%
Died	0	1, 2.2%	1, 1.6%
Euthanized	0	2, 4.4%	2, 3.3%
Transferred for ongoing care	0	2, 4.4%	2, 3.3%

Q1–Q3, first to third quartiles; Min–Max, minimum and maximum values within the group.

All but one dog ingested iron EDTA molluscicide, while one dog ingested a plant supplement containing iron EDTA. The median dose of elemental iron from iron EDTA snail pellets was 142.9 mg/kg (Q1–Q3 99.38–216.27, Min–Max 0.37–821.05), while the lowest dose at which clinical signs occurred was 31.2 mg/kg of elemental iron. The plant supplement ingested by a single dog contained 17% iron EDTA powder, but the amount ingested was unknown.

The majority of cases (62.3%) were primary accession; secondary and tertiary accessions were generally referred for desferoxamine chelation therapy. Time from ingestion to first presentation to a veterinarian varied for those cases in which it could be determined (*n* = 60) (Table 1). For the cases that were referred (*n* = 23), most had emesis induced before referral (18, 78.3%), three had enemas (13%), one (4.3%) was administered activated charcoal, and one (4.3%) had gastric lavage for decontamination before referral. Other treatments were given in low numbers of cases prior to referral; gastroprotectants in 5 dogs (21.7%), antiemetics in 2 (8.7%), and analgesia in 1 (4.3%).

Only 24.6% had witnessed ingestion of an iron-containing product, while in 47.5% the owners found an empty or disturbed packet of an iron-EDTA-containing product. High proportions of cases also had vomitus, gastric lavage fluid, diarrhea/feces, enema, or colonic lavage fluid consistent with iron EDTA ingestion (Table 1). Radiographs were not used for diagnosis in any cases.

Twenty dogs (32.8%) had no clinical signs before presentation to the referral hospital. Of the 41 dogs with clinical signs, vomiting without blood was the most common. The numbers and proportions of dogs with different clinical signs are displayed in Table 2, both before presentation and in-hospital. Thirty dogs (49.2%) had no abnormalities on physical examination. Of the 31 dogs with abnormalities, abdominal pain was most common (20/31, 66.7%), followed by dehydration (10/31, 32.3%), vasoconstrictive shock (6/31, 19.4%), pyrexia (3/31, 9.7%), and abdominal distention (3/31, 9.7%). Median heart rate was 120 beats/min (Q1–Q3 104–139, Min–Max 56–196). Respiratory rate was missing or described as panting for 27 dogs. Of those in which a numerical respiratory rate was recorded, the median was 28 breaths/min (Q1–Q3 24–32, Min–Max 15–44). Rectal temperature was reported for 51 dogs with a median of 38.6°C (Q1–Q3 38.25–38.85, Min–Max 37.5–39.9).

**Table 2:** Clinical signs reported before presentation and in-hospital among 61 dogs evaluated for iron EDTA ingestion.

Clinical signs	Number and proportion of dogs displaying clinical signs	
	Before presentation	In hospital
None	20 (32.8%)	21 (34.4%)
Vomiting without blood	21 (34.4%)	2 (3.3%)
Lethargy	16 (26.2%)	
Diarrhea without blood	15 (24.6%)	21 (34.4%)
Ataxia/weakness	12 (19.7%)	
Signs consistent with abdominal pain	10 (16.4%)	
Diarrhea—rust colored	5 (8.2%)	
Trembling	6 (9.8%)	
Collapse	3 (4.9%)	
Diarrhea with hematochezia or melena	3 (4.9%)	15 (24.6%)
Vomiting with hematemesis or coffee grounds appearance	2 (3.3%)	3 (4.9%)
Inappetence	2 (3.3%)	
Formed feces—rust colored, with snail pellets	2 (3.3%)	
Formed feces with blood	1 (1.6%)	1 (1.6%)

When considering the stage of iron toxicoses, 44 of 61 dogs (72.1%) had evidence of Stage 1 clinical signs, 4 dogs (6.6%) had evidence of Stage 2 clinical signs, and 19 dogs (31.1%) had Stage 3 clinical signs. No dogs had Stage 4 clinical signs (delayed gastrointestinal stricture). Additionally, 16 dogs never developed clinical signs (26.2%).

Serum iron concentrations are summarized in Table 1. Of the 41 dogs that had pre-chelation serum iron concentration measured, 29 (63%) had concentrations above the laboratory RI, while 12 (26.1%) were within the RI. Of the 21 dogs that had post-chelation serum iron measured, three (14.3%) were above the RI, 10 (47.6%) were within, and eight (38.1%) were below the RI. Where both pre-chelation and post-chelation iron concentrations were measured (*n* = 16), post-chelation concentrations were numerically lower than pre-chelation concentrations in all dogs.

Biochemistry profiles were performed in 33 dogs (54.1%). Of these, 23 dogs had a single biochemistry, 8 dogs had two biochemistry profiles, and 2 dogs had three biochemistry profiles performed during hospitalization. In the first biochemistry, 2 of 33 dogs (6.1%) had increased alanine aminotransferase (ALT) concentrations, 2 dogs had increased alkaline phosphatase (ALP), and 1 dog had hyperbilirubinemia. One of the five dogs with increased ALT had persistently increased ALT on serial biochemistry and later developed hepatic failure. One dog had mild azotemia, which was assessed to be pre-renal due to concurrent hemoconcentration. However, a urine-specific gravity was not performed to confirm this, nor was repeat biochemistry after IV fluid therapy.

Forty dogs (65.6%) underwent some form of gastrointestinal decontamination at the study hospital. This included emesis with apomorphine (30), emesis with soda crystals (7), colonic lavage (11), gastric lavage (2), and enema administration (2). Decontamination was successful in 31 of 38 dogs (81.6%) for which success was reported. Three dogs received activated charcoal: one with sorbitol, and two without.

Ten dogs were managed as outpatients (16.4%). For the 51 dogs managed as inpatients, treatments included IV fluids with isotonic crystalloids (44, 86.3%), desferoxamine (43, 84.3%), gastroprotectants (41, 80.4%), antiemetics and/or prokinetics (36, 70.6%), analgesia (24, 47.1%), and hepatoprotectants (5, 9.8%). Some dogs also received anxiolytics (14, 27.5%). Other treatments included oral lactulose (3, 4.2%), and one case each received vitamin C, an antihistamine, and antibiotics. Only 15 of the 41 dogs (36.6%) treated with gastroprotectants had evidence of gastrointestinal ulceration or erosion either before presentation or in the hospital (as evidenced by frank or digested blood in vomitus or feces). Table 3 includes more information about in-hospital treatment.

**Table 3:** Numbers and proportions of dogs receiving different types of medications following iron EDTA ingestion, separated by administration in-hospital or instructions for use at home after discharge.

Medication class	Medications administered in hospital (*n* = 51 treated as inpatients)	Medications prescribed for at-home use after discharge (*n* = 56 survived to discharge)
Gastroprotectants	In-hospital = 41/51, 80.4% Pantoprazole IV (26/41, 63.4%) Sucralfate PO (18, 43.9%) Omeprazole PO (8, 19.5%) Ranitidine PO (10, 24.4%)	To-go home = 35/56, 62.5% Omeprazole (30/35, 85.7%) Sucralfate (14, 40.0%) Ranitidine (3, 8.6%) Misoprostol (1, 2.9%)
Antiemetics and/or prokinetics	In-hospital = 36/51, 70.6% Maropitant (21/36, 58.3%) Metoclopramide (19, 52.8%) Ondansetron (6, 16.7%) Erythromycin (1, 2.8%)	To-go home = 9/56, 16.1% Maropitant (6/9, 66.7%) Ondansetron (3, 33.3%) Metoclopramide (1, 11.1%)
Analgesia	In-hospital = 24/51, 47.1% Methadone (11/24, 45.8%) Buprenorphine (10, 41.7%) Fentanyl CRI (4, 16.7%) Tramadol (3, 162.5%) Ketamine CRI (2, 8.3%) Oral lignocaine (1, 4.2%)	To-go home = 2/56, 3.6% Tramadol (1) Codeine (1)
Hepatoprotectants	In-hospital = 4/51, 7.8% S-AME with Silybin (2/4, 50%) S-AME alone in (1, 25%) S-AME, Silybin, and vitamin E (1, 25%)	To-go home = 4/56, 7.1 % S-AME with Silybin (2/4, 50%) S-AME alone in (1, 25%) S-AME, Silybin, and Vitamin E (1, 25%)

S-AME, S-Adenosylmethionine.

Of the 43 dogs who received desferoxamine, the protocol for administration was either as an IV CRI at 15 mg/kg/h (27/43, 62.8%), 40 mg/kg IM q8h (6, 14%), 40 mg/kg IM q4h (3, 7%), 40 mg/kg IM q6h (2, 4.7%), or 40 mg/kg IV q6h (2, 4.7%), while 3 dogs had other protocols. The median duration of desferoxamine CRI was 15 h (Min–Max 2.5–48, Q1–Q3 10.25–19.75). Of those receiving intermittent desferoxamine dosing, two dogs had a single dose, one dog had two doses, three dogs had three doses, four dogs had four doses, and two dogs had five doses. For those receiving intermittent dosing, the desferoxamine dose received in the first 24 h of treatment varied, with a median of 120 mg/kg (Min–Max 40–333 mg/kg).

While the reason for clinicians choosing to treat with desferoxamine was not always evident from retrospective record review, the estimated ingested dose of elemental iron was higher, when known, in the dogs treated with desferoxamine (156 mg/kg, Min–Max 70.91–821.05), than those not treated with desferoxamine (73.13 mg/kg, Min–Max 0.37–173.98) (*p* = 0.006). While serum iron concentrations were not known to the clinicians when starting desferoxamine, median pre-chelation serum iron concentrations, when measured, were also higher in the dogs that received desferoxamine (66.3 μmol/L, Min–Max 9–356) than those that did not (23 μmol/L, Min–Max 11–57) (*p* < 0.001).

Seven dogs that received desferoxamine ultimately had pre-chelation serum iron concentrations within RI. Criteria used to guide cessation of chelation therapy included serum iron concentrations (18/43, 41.9%), urine color (11, 25.6%), adverse reaction to desferoxamine (2, 4.7%), or was not reported in the medical record (12, 27.9%).

Twenty dogs were sent home with no ongoing treatment. Table 3 includes more information about gastroprotectant, antiemetic, and/or prokinetic, analgesic, and hepatoprotectant medications in-hospital and continued after discharge. In addition to medications outlined in Table 3, two dogs were discharged with amoxicillin-clavulanic acid; one for treatment of bacterial cystitis and the other for febrile neutropenia. Another dog was sent home with metronidazole as part of the treatment regimen for concurrent inflammatory bowel disease.

Overall, 56 of 61 (91.8%) dogs survived to discharge home, 2 (3.3%) were transferred to another veterinarian for ongoing care, 2 (3.3%) were euthanized, and 1 died naturally. One of the dogs that initially survived to discharge was euthanized 2 weeks later, after developing a severe acute hepatopathy.

Five dogs had notable complications of iron toxicity or its treatment, including anaphylactic reactions to an accidental bolus of desferoxamine (2), allergic reactions (2), febrile neutropenia (1), and cardiopulmonary arrest (CPA) (1). Two dogs also had complications secondary to diarrhea (lower urinary tract infection and perineal fecal scalding), and one dog required re-admission after discharge for ongoing treatment of gastrointestinal upset.

The first dog that developed an anaphylactic reaction to desferoxamine was a 9-year-old male neutered Dachshund that ingested 139 mg/kg of elemental iron (iron EDTA molluscicide). He had been on a 15-mg/kg/h desferoxamine CRI for 12 h when he received an inadvertent 100 mg (1 mL) bolus of desferoxamine over 2 min when the syringe driver was being refilled. The dog immediately vomited, became stuporous, developed injected mucous membranes, hypotension, gall bladder wall edema, and free abdominal fluid on point-of-care ultrasound (POCUS). This dog responded well to discontinuation of desferoxamine and aggressive treatment with IV fluid boluses and adrenaline. Two 10-mL/kg IV isotonic crystalloid boluses were administered, 10 min apart. Adrenaline was dosed at 10 μg/kg IM, followed by 1 μg/kg IV 10 and 15 min later, followed by a CRI at 0.01 μg/kg/min for 1 h. With these treatments, cardiovascular instability resolved, and the dog survived to be discharged the following day.

The second dog that had an anaphylactic reaction was an 8.5-year-old male neutered mixed-breed dog that had ingested an unknown quantity of iron EDTA molluscicide. He was also treated with 15 mg/kg/h desferoxamine CRI when a small volume was accidentally flushed down the line. This resulted in immediate vomiting, collapse with tachycardia, hypotension, and tachypnea. Hypotension (systolic blood pressure 70–90 mmHg), tachycardia (HR 180–190 bpm) and hyperlactatemia (lactate 7.5 mmol/L, RI 0.5–2.0 mmol/L) persisted despite treatment with IV boluses of balanced isotonic crystalloids totaling 45.9 mL/kg, chlorpheniramine IM, and maropitant, prompting the administration of low-dose adrenaline (0.005 mg/kg) IV. Adrenaline administration resulted in rapid resolution of the tachycardia and hypotension, with a brief period of undefined arrhythmia and hypertension, before normalization of cardiovascular parameters. No further desferoxamine was given, and the dog was discharged the following day.

Two dogs had possible allergic reactions to desferoxamine. A 10-month-old Fox terrier developed ventral erythema, generalized urticaria, and pruritus, 8 h after commencing a desferoxamine CRI (15 mg/kg/h). The signs resolved on cessation of desferoxamine, after which the CRI was restarted at a lower concentration for a further 9 h without recurrence. This dog had a history of environmental allergies. Another dog, a 7-year 5-month-old female spayed American Staffordshire bull terrier, developed generalized urticaria and facial angioedema 4 h after commencing a 15-mg/kg/h desferoxamine CRI. This dog had a known grass allergy and was walked outside just before the allergic reaction. Her symptoms resolved with chlorpheniramine injection and discontinuation of the desferoxamine CRI, and did not recur when the desferoxamine CRI was restarted for another 6 h. This dog survived to initial discharge but later developed hepatic failure and is described below.

The dog with febrile neutropenia, a 2-year 4-month-old female spayed Australian shepherd, was diagnosed with sepsis, suspected to be of gastrointestinal origin based on fulfillment of systemic inflammatory response syndrome (SIRS) criteria (temp 40.2°C, heart rate 138 bpm, neutrophils 2.89 k/μL [3–11.5]) after approximately 48 h of hospitalization. She had ingested iron EDTA molluscicide (88 mg/kg of elemental iron) and developed lethargy, ptyalism, and diarrhea, which continued over the first 3 days of hospitalization. No other inflammatory or infectious focus was found on repeat physical examination, POCUS (abdominal and thoracic), hematology, biochemistry, urinalysis (including culture), or inspection (and changing) of the IV catheter site. Commencement of amoxicillin-clavulanic acid (30 mg/kg IV q6h) resolved the fever within 8 h and the neutropenia by the next day. This dog survived and was discharged to complete a 5-day antimicrobial course.

Given the scarcity of reports of fatal iron toxicosis in dogs, more information is provided about the non-survivors. The one dog that died during hospitalization was an 11-year 9-month-old female spayed Border collie that was referred 12–24 h after ingesting iron EDTA molluscicide (~110 mg/kg of elemental iron). Decontamination was not performed. Despite normal vital signs at presentation, she had evidence of a mild free water deficit (Na 154 mmol/L, RI 140–150 mmol/L), a mild mixed acid–base disorder (metabolic acidosis and respiratory alkalosis), and an increased serum iron concentration (89.7 μmol/L [RI 15.0–41.7]). Chelation therapy was administered with a desferoxamine CRI (15 mg/kg/h) for approximately 38 h, and other supportive treatments were provided. About 24 h after ingestion, the dog was noted to have monomorphic accelerated idioventricular rhythm (AIVR) with occasional pulse deficits and a heart rate up to 160 bpm. On the third day of hospitalization, low-volume watery diarrhea and AIVR continued. Body temperature was 39.1°C. Hematology revealed a marked neutropenia (segmented neutrophils 0.58 k/μL [RI 3–11.5], bands 0.01 k/μL, mild toxic changes), a mild normocytic normochromic anemia (Hgb 116 g/L [RI 120–180]), and the presence of nucleated red blood cells (9/100 white blood cells). Antimicrobial coverage was provided with amoxicillin-clavulanic acid (30 mg/kg IV q 6 h). Concurrently, the biochemistry profile revealed worsening of systemic inflammation; the C reactive protein (CRP) had increased to 115 mg/L from 73 mg/L the day prior (RI < 10). Mild azotemia also developed; the creatinine increased to 133 μmol/L (RI 44–132) from 115 μmol/L the day prior. Approximately 55 h after ingestion, the dog became mentally obtunded, tachycardic (HR 160 bpm), and hypotensive (SBP 70 mmHg). Shock continued despite two 10-mL/kg IV boluses of isotonic crystalloid, and while a noradrenaline infusion was being prepared, the dog experienced bradycardia progressing to CPA. Initial cardiopulmonary resuscitation achieved return of spontaneous circulation within 2 min, but repeated CPA occurred within minutes, and CPR was not attempted a second time at the owner’s request.

Of the two dogs that were euthanized, one was euthanized for financial reasons, while the other dog was euthanized due to the development of acute respiratory distress syndrome (ARDS). The dog that developed ARDS was an 8-year-old female intact bull Arab that had ingested ~146 mg/kg elemental iron (as iron EDTA molluscicide). Despite successful induction of emesis within 1–2 h of ingestion, the dog continued to have vomiting (with coffee grounds appearance) and to produce feces containing iron material up to 36 h after ingestion. Pre-chelation serum iron was increased at 72.3 μmol/L (RI 15.0–41.7). Chelation therapy with desferoxamine at 15 mg/kg/h IV CRI for 45 h, and supportive treatment were provided. Post-chelation serum iron was low (12.8 μmol/L). The dog developed acute onset tachypnea 72 h post-iron ingestion, approximately 4 h after discontinuation of the desferoxamine CRI. This progressed to dyspnea over the next 8 h, requiring mechanical ventilation. Thoracic radiographs, computed tomography (CT), or bronchoalveolar lavage were not performed before the patient was euthanized; however, three out of four of the VetALI/VetARDS criteria were met (acute onset of tachypnea and labored breathing at rest, known risk factors [drugs or toxins], and evidence of inefficient gas exchange [hypoxemia without positive end expiratory pressure and known FiO_2_]) (24). Post-mortem examination was not performed.

The aforementioned dog that initially survived to discharge but was euthanized 2 weeks later was a 7-year 4-month-old American Staffordshire bull terrier dog that ingested iron EDTA molluscicide (162.5 mg/kg elemental iron). Her ALT was mildly increased on presentation (135 U/L, RI 10–125), but due to financial constraints, the owner declined repeat ALT measurement during hospitalization. Nonetheless, she underwent gastrointestinal decontamination and received desferoxamine and supportive care as an inpatient for 1 day. The dog re-presented 12 days later to the study hospital for lethargy and hematemesis. Physical examination revealed severe vasoconstrictive shock. Biochemistry revealed markedly increased liver enzymes, including ALT above the measurable limit and ALP at 1,356 U/L (RI 20–150). Coagulation times (prothrombin time and activated partial thromboplastin time) were prolonged above the upper limit of detection, indicating a severe coagulopathy. The dog was subsequently euthanized.

## 4 Discussion

This multicenter retrospective case series describes the clinical course and treatment of dogs following iron EDTA ingestion. Gastrointestinal signs predominated, and the reported dog cases had a clinical course somewhat consistent with the first three stages of progression of iron toxicosis described in humans; however, many cases remained asymptomatic, and the delayed fourth stage of the toxidrome was not observed. Additionally, for two of the non-survivors in our case series, the causes of death (cardiovascular collapse and hepatic failure) reflect the two most common causes of death due to iron toxicosis in humans (25). Since most dogs presented as primary accession cases, our results are likely to reflect cases seen in primary care practices, despite our study centers being emergency and referral hospitals.

In this study, approximately one-third of dogs never developed clinical signs of iron toxicosis, likely due to a combination of low-dose ingestion, early presentation to veterinary hospitals for decontamination, and prompt commencement of therapy, including chelation. The remaining two-thirds of dogs developed early gastrointestinal upset, with smaller proportions having a period of apparent recovery and going on to develop systemic signs. The predominance of gastrointestinal signs mirrors the findings of previous reports of iron toxicosis in dogs (7, 8, 12–14). Early gastrointestinal signs occur during Stage 1 toxicosis when ingested free iron promotes free-radical formation and oxidative damage in the gastrointestinal mucosa, and iron may have a direct corrosive effect on the gastrointestinal mucosa. Severe gastrointestinal fluid loss likely resulted in dehydration and vasoconstrictive shock seen in a portion of the dogs described herein.

The occurrence of pyrexia in three dogs in this case series has not previously been reported in dogs (7) but is consistent with findings in some humans, where systemic inflammation occurs as a result of gastrointestinal mucosal damage (5, 26–31). One dog was diagnosed with sepsis. This dog and another dog also developed neutropenia during hospitalization. While neutrophil consumption in sepsis is one differential diagnosis, drug-induced neutropenia as an idiosyncratic reaction to desferoxamine is another consideration. While desferoxamine-induced neutropenia has not been reported in dogs, it is known to occur in humans, as is neutropenia secondary to other iron chelators (32).

The frequency of ataxia, weakness, and collapse was unexpected in dogs following iron ingestion, but consistent with some dogs having reluctance to move and neurologic abnormalities in the study by Lauinger and colleagues (7). The mechanism of these abnormalities remains unclear, but it may be in response to severe abdominal pain or the effects of iron toxicosis on the nervous system. Neurologic signs in humans are also described in severe cases of iron poisoning and remain poorly understood (33, 34).

Four dogs in our study were considered to have Stage 2 toxicosis, in that they had early clinical signs, were then asymptomatic and normal on examination at presentation to the study hospital, followed by re-development of more severe symptoms later. In humans, if symptoms do not develop within 6 h of ingestion, clinical toxicity is unlikely to occur (5, 30). However, given that signs could be missed in our veterinary patients and Stage 2 toxicosis could be misinterpreted as lack of toxicosis, it may be prudent that any dogs presenting with a history of exposure to iron EDTA products have serum iron concentration measured, be monitored for up to 30 h post-ingestion, and possibly even have empirical chelation therapy if estimated ingested dose is high despite being asymptomatic on presentation.

The majority of dogs that had Stage 3 toxicosis had ongoing gastrointestinal signs, with or without cardiovascular instability and metabolic derangement. As most symptomatic dogs received fluid therapy with isotonic crystalloids and chelation with desferoxamine, it is likely that Stage 3 toxicosis was abated with treatment, and may have been underestimated. Indeed, some classification schemes in humans include five stages, where Stage 4 is the clinical recovery beginning soon after the initiation of fluid and chelation therapy (6). The dog in our case series with the most severe signs of Stage 3 toxicosis died in the hospital despite aggressive treatment. Cardiovascular collapse in iron toxicosis occurs due to iron-induced vasodilation and myocarditis, resulting in negative inotropy. Iron can also stimulate the release of ferritin, serotonin, and histamine, which induce vasomotor collapse (5, 25, 30, 31, 35, 36). Although this dog received chelation therapy, the iron load in the heart may decrease less rapidly than in organs such as the liver, as occurs with humans (37). An alternative explanation could be sepsis-induced cardiovascular dysfunction given the concurrent pyrexia, neutropenia, and increased CRP.

The dog that developed liver injury and was euthanized was not considered a classic Stage 3 toxicosis since liver failure was identified beyond the timeframe when Stage 3 toxicosis is expected. However, some authors describe five stages of iron toxicosis (5), including liver injury in Stage 4 (2–5 days following ingestion), and cirrhosis in Stage 5 (2–6 weeks following ingestion), which better aligns with the clinical course in this dog. While iron toxicosis was the suspected cause of liver failure, the lack of diagnostic investigation precludes a definitive conclusion as to causation. Additionally, the dog in our case series received trazodone as an anxiolytic during hospitalization, and a case of suspected trazodone-induced hepatotoxicity has been reported in a dog (38). Thus, it is unclear if this patient developed hepatic failure due to iron toxicosis, trazodone-induced hepatotoxicity, or another hepatic insult.

As the liver is the first organ to receive portal blood containing toxic amounts of iron, absorption of iron in the reticuloendothelial system is rapid. High metabolic activity also predisposes the liver to free-radical injury (5, 29–31, 39). The case report by Brutlag and colleagues described a dog who developed mildly increased ALT (175 U/L) after ingestion of two packets of oxygen absorber, which reduced to 136 U/L 20 h later but increased back to 217 U/L 3 months later. Although this dog was eventually lost to follow-up, the dog was clinically well upon initial recheck (14). In humans, the incidence of iron-induced hepatotoxicosis is low, and clinically significant hepatotoxicosis is rare when serum iron concentration is less than 125.3 μmol/L (5, 25, 35). However, there have been reports of clinically significant hepatotoxicosis at lower serum iron concentration; thus, it is unclear if iron-induced hepatotoxicosis is dose-dependent or idiosyncratic (25, 35, 39–41). Pre-chelation serum iron concentration was not available from the dog in our study, and was 78.04 μmol/L in the other reported case (14). While no cases developed delayed gastrointestinal signs consistent with Stage 4 toxicosis, many of the cases were lost to follow-up, precluding any conclusions about the occurrence of this stage.

The median dose of elemental iron in our study was slightly higher than in the study by Lauinger and colleagues (142.91 vs. 122 mg/kg), but both were estimates (7). The median serum iron concentration was similar in the two studies, although the maximum serum iron concentration was higher in our case series. High exposure to elemental iron in dogs ingesting molluscicide could be due to the misleading “pet friendly” labeling on the iron EDTA snail pellets packaging, resulting in owners not keeping them away from pets. While two experimental studies in dogs showed that 225–250 mg/kg of elemental iron intraduodenally or via enema was fatal in dogs (42, 43), most dogs in our case series survived estimated exposures this high with appropriate treatment. As with many studies of toxin ingestion in dogs, quantities consumed were estimates, limiting conclusions that can be drawn about dose-effect relationships.

Prompt gastrointestinal decontamination in dogs in this case series undoubtedly mitigated the severity of the toxidrome in some dogs. Unsuccessful decontamination in some of the dogs could be due to prolonged time between ingestion and presentation, rapid absorption of iron products, or the stickiness of iron products, making them adhere to the gastrointestinal wall and/or stick together, forming iron bezoars (27–31, 36, 44–46). Activated charcoal was administered in a small number of cases, despite documentation that it does not bind to iron (5, 31, 47, 48). Based on human guidelines, the existing veterinary literature, and the results of this study, decontamination via emesis, gastric lavage, and enema should be performed in cases of recent ingestion to prevent further absorption of iron, but administration of activated charcoal is not recommended.

The majority of dogs showing symptoms were treated as inpatients, although only for a short duration of hospitalization. Most received IV fluid therapy and desferoxamine, and many received gastroprotectants, antiemetics, prokinetics, and analgesia. Such a treatment approach is similar to that of human guidelines that recommend first prioritization of fluid balance and prevention of further iron absorption, followed by symptomatic care.

A similar proportion of dogs in this case series (43/61, 84.3%) received desferoxamine as in a recent large multicenter case series of Australian dogs with iron EDTA molluscicide ingestion (54/73, 74%) (2). This is not surprising given the high estimated doses of elemental iron ingested in both studies. General recommendations in veterinary toxicology textbooks regarding chelation therapy suggest its use in those experiencing severe toxicosis or if serum iron concentrations are greater than 54 μmol/L (300 mg/dL) (6). Unfortunately, serum iron concentrations are generally measured by laboratories external to veterinary clinics, resulting in a delay in results. As such, it is likely that veterinarians are choosing to administer desferoxamine in any cases where clinical signs are already present, and for those with ingestions of more than 60 mg/kg of elemental iron since this is reported to result in severe intoxication (3, 9). Indeed, all dogs in this case series that received desferoxamine had an estimated ingested dose, where known, of greater than 60 mg/kg.

The most commonly used dose for desferoxamine was a CRI at 15 mg/kg/h IV, which is also the most common recommendation in humans. In humans, the IM route can be painful, has been shown to have erratic absorption and lower efficacy compared to IV administration (5, 30, 31, 36, 47–50). Interestingly, pain or other adverse effects associated with intermittent IM injections were not noted in the medical records in our study. Overall, our study showed that desferoxamine appeared to be well tolerated when administered according to published protocols. Two dogs developed an anaphylactic reaction and hypotension due to an inadvertent bolus. In humans, hypotension represents the most well-known adverse effect and is thought to be due to histamine release, venous dilation, and reduced cardiac output. Hypotension does not tend to occur when administered at rates less than 15 mg/kg/h (5, 30, 36, 51). A rapid bolus of desferoxamine should therefore be avoided. Two dogs developed allergic reactions, although only one was suspected due to desferoxamine administration. Allergic reactions may occur due to contamination in the fermentation products derived from the purifying process of this siderophore from bacterial cultures (52).

One dog in our study developed acute dyspnea and clinical features consistent with ARDS. Similar to our case, ARDS has recently been reported in a dog receiving desferoxamine for iron ingestion in a recent case series (7). Risk factors for ARDS in our dog case include systemic inflammation, drugs (including desferoxamine), and toxins (including iron). Multiple cases of pulmonary toxicosis, including ARDS, have been reported as adverse effects of desferoxamine in humans (53, 54). As with many cases of ARDS, it was not possible to determine if lung injury occurred due to iron toxicosis, desferoxamine, or another cause such as aspiration. Regardless, clinicians should be aware of the potential for either of these to cause fatal pulmonary complications.

There was inconsistent documentation of when chelation therapy was stopped, and post-chelation serum iron concentrations and urine color were not monitored for all cases. One veterinary author recommends continuing chelation therapy until serum iron concentration falls below 53.7 μmol/L (3). In humans, no specific guidelines exist on when to discontinue chelation therapy. Some authors also recommend using serum iron concentration as an endpoint, but at a much lower value of 17.9 μmol/L (47). Unfortunately, using serum iron concentrations to guide therapy is often impractical in veterinary medicine when measurements are being performed at external laboratories, resulting in delays. Furthermore, serum iron concentrations can drop due to intracellular uptake of iron; therefore, low serum concentration does not equate to low tissue iron nor resolution of toxicosis. In addition, hemolyzed samples can falsely elevate serum iron concentrations (55), and measurement of serum iron with spectrophotometry can be inaccurate in the presence of desferoxamine. Nonetheless, the group median post-chelation serum iron concentration was lower than the median pre-chelation concentration, and each individual dog that had both pre-chelation and post-chelation measurements taken had lower post-chelation iron concentrations. This suggests that desferoxamine is an effective iron chelator in dogs, although it may be that serum iron concentrations would have reduced with time regardless of the administration of chelation therapy. Due to the retrospective nature of our study and unknown timing of serum iron measurements, we elected not to statistically compare pre- and post-chelation serum iron concentrations, or correlations between reduction in serum iron concentration and outcome. Such research questions would be best answered by a prospective, randomized clinical trial.

Some clinicians continue desferoxamine based on the severity of clinical signs and persistence of discolored urine. This is because when desferoxamine binds to iron, *a vin rose-colored* complex is formed and excreted in the urine. Urine discoloration suggests excretion of iron in the urine, while the disappearance of the *vin rose* color of the urine is suggestive that there is no further iron to be bound. These recommendations, however, did not arise from any data, and clear urine color has been found in patients with persistently high serum iron concentrations. It is overall recommended that chelation therapy be continued as long as clinical signs persist, and that patients be evaluated for recurrence of clinical signs 2–3 h after stopping chelation (5, 30, 31, 36, 47, 48, 50, 53).

Regarding other treatments, many dogs received gastroprotectants, while only five dogs received hepatoprotectants. Some dogs that received antacids in our case series lacked evidence of gastroduodenal ulceration or erosion. The American College of Veterinary Internal Medicine (ACVIM) consensus statement on the rational use of gastroprotectants suggests that gastroprotectants such as antacids should only be used in cases of documented gastroduodenal ulceration or erosion, identified based on clinical signs (hematemesis) or endoscopically; however, it does not specifically refer to iron toxicosis (56). It was likely that clinicians administered antacids prophylactically due to descriptions of gastric ulceration in cases of iron toxicosis, although the role of antacids prophylactically in this toxidrome is unknown and warrants further investigation. It may have also been difficult to differentiate between hematemesis and the red/rust color of iron in vomitus. Interestingly, administration of hepatoprotectants in humans with iron toxicosis is not well described, and recommendations cannot be made about whether hepatoprotectants should be used pre-emptively in patients without evidence of hepatotoxicity.

There were several limitations to our study. Some data were missing, and there was significant variation in diagnostic tests performed. Timing of measurement of serum iron concentrations varied, and precise timing was difficult to discern; hence, all we reported was simple classification as either pre- or post-chelation. Serum iron concentrations can be increased by factors other than excessive iron ingestion, including pre-analytic factors such as hemolysis, and other conditions, including hepatocellular necrosis or hypercortisolemia. Data were not routinely available to assess these factors, and thus, we cannot rule out the potential for falsely increased serum iron concentrations due to these factors in a subset of cases. Since follow-up was not standardized, we cannot make conclusions about the development of Stage 4 signs or other delayed complications of toxicosis. Similarly, while many dogs had good outcomes after chelation therapy, it is possible that they would have also had a good outcome without chelation therapy, and thus we cannot make conclusions regarding the efficacy of desferoxamine. Indeed, seven dogs that received desferoxamine had pre-chelation serum iron concentrations that were within RI, suggesting that they may not have benefited from chelation. Additionally, our study population is small, and the cohort may be biased toward more severely affected cases in which clinicians deem the need for serum iron concentration measurement and/or chelation therapy. Future prospective investigations should have a more consistent collection of standardized data, including the amount of iron (therefore elemental iron dose) ingested, serum iron measurements at standardized timepoints after ingestion, parameters such as venous blood gas and coagulation testing, and follow-up for up to 6 weeks to detect any occurrence of Stage 4 symptoms.

## 5 Conclusion

Our study showed that gastrointestinal signs similar to Stage 1 iron toxicosis in humans are common in dogs after iron EDTA ingestion, and some dogs go on to develop Stage 2 and 3 toxicoses. Most dogs tolerated desferoxamine chelation, but two dogs developed anaphylactic reactions after rapid IV injection, two developed neutropenia, and one developed ARDS. While the prognosis for iron ingestion and toxicosis in dogs is good when prompt and appropriate treatment is instituted, veterinarians should be aware of the potential for iron toxicosis to result in cardiovascular collapse and hepatic failure. Further prospective studies of naturally occurring iron toxicosis in dogs are warranted to improve our understanding of the toxidrome.

## Data Availability

The raw data supporting the conclusions of this article will be made available by the authors, without undue reservation.
